# Peptides With Triplet-Tryptophan-Pivot Promoted Pathogenic Bacteria Membrane Defects

**DOI:** 10.3389/fmicb.2020.00537

**Published:** 2020-04-09

**Authors:** Shuli Chou, Qiuke Li, Zaitseva Nina, Lu Shang, Jiawei Li, Jinze Li, Zhihua Wang, Anshan Shan

**Affiliations:** Institute of Animal Nutrition, Northeast Agricultural University, Harbin, China

**Keywords:** triplet-tryptophan-pivot peptides, probiotic-ineffective, long-term protection, low drug resistance, *in vivo* application

## Abstract

Development of probiotic-ineffective antimicrobial peptides (AMPs)-based coatings that can kill pathogenic bacteria at low concentrations but are essentially harmless (even high concentrations) to probiotic organisms is a relatively new trend for therapy against GI tract infections. In this study, a series of triplet-tryptophan-pivot peptides with various hydrophilic amino acids was constructed. One AMP in particular, S7, showed bactericidal activity against *Staphylococcus epidermidis*, *Pseudomonas aeruginosa*, *Escherichia coli* and antibiotic-resistant *Staphylococcus aureus*, yet was shown to be harmless to *Lactobacillus rhamnosus*, a key GI tract commensal. Furthermore, antibacterial mechanism assays, drug resistance assays, and mouse model tests suggested that S7 was useful in a clinical setting as it proved to significantly reduce bacterial load and cytokines (TNF-α, IL-6; *P* < 0.05) with a low probability of resistance via bacterial membrane physical destruction and formation of intracellular ROS. Combined, the results show that a triplet-tryptophan-pivot peptide containing a pair of serine residues was an excellent pathogen-selective candidate for medical devices and was potentially useful in food preservation, crop protection, and human health.

## Introduction

The unceasing proliferation of pathogenic resistance among key human GI pathogens, for example, the appearance of drug-resistant *Staphylococci*, *Enterococci*, *Klebsiella pneumoniae*, and *Pseudomonas* spp., leads to prolonged illness and a risk of death (Tenover, 2006). Moreover, excessive application of antibiotics has further selected for vertical and horizontal dissemination of many antibiotic resistance markers across multiple strains and species. Thus, it is essential to find new antimicrobial agents that are not affected by traditional mechanisms of bacterial resistance. Antimicrobial peptides (AMPs) often behave like hormones or even as medicines with antimicrobial, antihypertensive, immunomodulatory, opioid, and antioxidant activities. For example, alpha-melanocyte stimulating hormone (α-MSH), a short cationic peptide, was a strong potential anti-infective agent (Singh and Mukhopadhyay, 2014). Additionally, 60 therapeutic peptides have been approved by US Food and Drug Administration (FDA) as antibodies, vaccines, and antimicrobial agents, etc. (Aguilar-Toalá et al., 2019). These peptides provide microbicidal activity via physical disruption of a pathogen’s membrane and subsequent leakage of cytoplasmic components, which greatly reduces the development of bacterial resistance because it is metabolically costly for the bacteria to mutate or repair the membrane’s molecular constituents (Makovitzki et al., 2008; Wimley, 2010; Ong et al., 2013; Ma et al., 2016; Jiang et al., 2017; Dong et al., 2019). Thus, AMPs can also be widely used in agriculture and the food industry to replace the long-term and indiscriminate use of pesticides and antibiotics (Zanutto-Elgui et al., 2019). Probiotics, such as lactic acid bacteria (LAB) or bifidobacteria, have tremendous potential for development of healthy diets, treatment, and prevention (Bubnov et al., 2018). Thus, pathogen-specificity is a major property, in addition to high resistance to degradation, low toxicity toward the host and low production cost, for next-generation antimicrobials (Fuente-nunez et al., 2017; Torres et al., 2019). Since *Staphylococcus epidermidis*, *Staphylococcus aureus*, *Escherichia coli*, and *Pseudomonas aeruginosa* are all prevalent pathogens in implant-associated infections (Barbosa et al., 2019), peptides with activity against these pathogens instead of probiotics have great potential.

It is well-known that sequence parameters such as amino acid frequency, hydrophobicity, charge ratio, structure could be modified to achieve ideal functions (Shao et al., 2019). Furthermore, the previous research demonstrated that composition of amino acids largely accounted for the antimicrobial spectrum in an active template (Mishra and Wang, 2012; Zhu et al., 2014). Thus, tryptophan, which showed a real ability to enhance peptide aggregation in solution and on a bilayer surface via π–π interactions and to increase membrane insertion and disruption, was first selected to ensure hydrophobicity (Zhao et al., 2013; Chou et al., 2016). Additionally, Zarena et al. (2017) reported that a Trp triplet stabilized by both aromatic-aromatic and aromatic-aliphatic interactions, shown as a WWW motif, can be applied to generate bacteria-targeting sequences. Following this Trp motif, a positive-charged amino acid arginine (Arg), which causes toroidal pore defects in the anionic membrane and positively influences electrostatic interactions between peptides and the negatively charged bacterial membrane surface (Chou et al., 2016), was added to obtain amphipathic sequences. Our research indicated a vital role of the hydrophilic amino acid to adjust the antibacterial spectrum but its exact effect was not distinguished (Chou et al., 2019). Finally, the resulting primary peptide structure simplified as RRWyWWWyWRR, where y represents an uncharged hydrophilic amino acid (Schema 1) was proposed as a probiotic-neutral sequence. All peptides were amidated to increase their stabilization and net charge. The overall objective of this study was to facilitate the development of peptide-based synthetic strategies to generate selective-function bioactive agents.

**SCHEMA 1:**
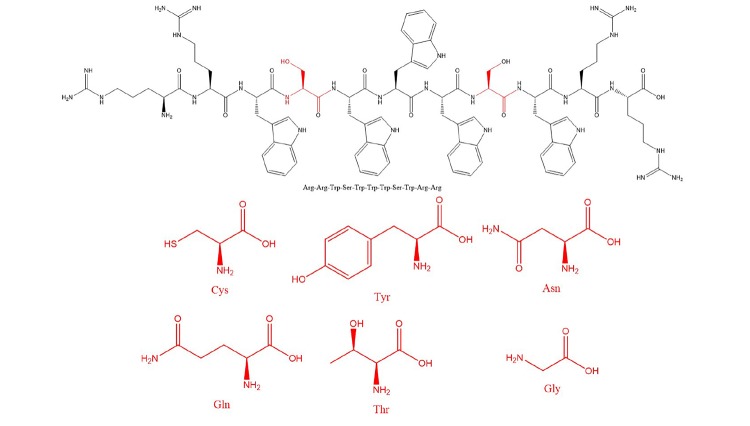
General schematic of designed peptides.

## Materials and Methods

### Materials

Mueller-Hinton broth (MHB), Mueller-Hinton agar (MHA) and MRS powder were obtained from AoBoX (China). Bovine serum albumin (BSA), Triton X-100, polymyxin B, 4-(2-hydroxyethyl)piperazine-1-ethanesulfonic acid (HEPES), dimethyl sulfoxide (DMSO), BODIPY-TR-cadaverine (BC), 3-(4,5-dimethylthiazol-2-yl)-2,5-diphenyltetrazolium bromide (MTT), 3,3′-dipropylthiadicarbocyanine (disc_3__–__5_), ethanol (analytical grade, 99%), tertiary butanol (analytical grade, 99%), acetone (analytical grade, 99%), glutaraldehyde (synthetic grade, 50% in H_2_O), lipoteichoic acid (LTA) from *S. aureus* and lipopolysaccharide (LPS) from *E. coli* were purchased from Sigma-Aldrich (China). Phosphate-buffered saline (PBS) solution, sodium chloride, potassium chloride, ammonium chloride, calcium chloride, zinc chloride, magnesium chloride, and ferric chloride were purchased from Kermel (China). Glucose (analytical grade) was obtained from Zhiyuan (Guangdong, China). DMEM phenol red-free medium and fetal bovine serum were purchased from Gibco (Beijing, China). All these reagents were used according to the required concentration range.

### Bacterial Strains

The bacteria strains *Escherichia coli* ATCC 25922, *Pseudomonas aeruginosa* ATCC 27853, *Staphylococcus aureus* ATCC 29213, *Escherichia coli* UB 1005, methicillin-resistant *Staphylococcus aureus* (MRSA) ATCC 43300 and *Staphylococcus epidermidis* ATCC 12228 were obtained from the College of Veterinary Medicine, Northeast Agricultural University. *L. plantarum* 8014, *Lactobacillus rhamnosus* 1.0386, *L. rhamnosus* 7469, *L. rhamnosus* 1.0911, *L. rhamnosus* 1.0385, and *L. rhamnosus* 1.0925 were obtained from the Key Laboratory of Food College, Northeast Agricultural University.

### Peptide Synthesis and Sequence Analysis

The peptides designed in this study were synthesized by Sangon Biotech, Co., Ltd. (Shanghai, China), and the molecular masses of these peptides were determined using matrix-assisted laser desorption/ionization time-of-flight mass spectrometry (MALDI-TOF MS; Linear Scientific, Inc., United States), with α-cyano-4-hydroxycinnamic acid as the matrix. The purity of the peptides (95%) was assessed by reversed-phase high-performance liquid chromatography (HPLC) using a Shimdzu Inertsil ODS-SP column (4.6 mm × 250 mm, 214 nm, 20 μL) and a non-linear water/acetonitrile gradient containing 0.1% trifluoroacetic at a flow rate of 1.0 mL/min.

### Cytotoxicity Measurements

The cytotoxicity of the peptides was determined with three cell types, namely, fresh, healthy human erythrocytes (obtained from a healthy donor Jiawei Li); murine macrophage cell line RAW264.7 cells; and human embryonic kidney (HEK) 293T cells, via hemolysis and MTT dye reduction assays. The release of hemoglobin and MTT dye reduction were monitored by measuring the absorbance at 570 nm. The tests were performed at least three times.

### MIC Measurements

The antibacterial activity of the peptides was measured using a method adopted from the Clinical Laboratory Standards Institute (CLSI), with modifications. 50 μL bacterial cells solution with 1000-fold of OD_600_ = 0.4 was mixed with 50 μL of each peptide of various concentration ranged from 2 to 64 μM dissolved in BSA (0.2% with 0.01% acetic acid) and incubated in sterile 96-well plates at 37°C for 24 h. The MICs were calculated as the lowest concentration of peptide with no microbial growth being observed. Broth with microbial cells and uninoculated broth were used as positive control and negative control, respectively. The tests were performed at least three times.

### Salt and Serum Sensitivity Assay

Different final concentrations of physiological salts (150 mM NaCl, 4.5 mM KCl, 6 μM NH_4_Cl, 8 μM ZnCl_2_, 1 mM MgCl_2_, and 4 μM FeCl_3_) and serum (50, 25, 12.5%) was mixed into Mueller-Hinton broth (MHB) to determine sensitivity of peptides to the salt and serum against *E. coli* ATCC 25922 and *S. aureus* ATCC 29213. The subsequent steps were consistent with the MIC determination method.

### Timed Killing Curve and Fractional Survival Tests

The ability of peptides to kill bacterial cells was further investigated by analyzing the fractional cell survival upon peptide treatment at various exposure times to show the dramatic differences in killing speed between comparable peptides. Briefly, *E. coli* ATCC 25922 or *S. aureus* ATCC 29213 (0.5–1 × 10^6^ CFU/mL) was treated at the 1/2, 1, and 2 MIC of peptides. At various time periods (0, 5, 10, 30, 60, 120 min), microbial suspensions were diluted 10- and 100-fold, and then 50 μL diluted suspension was plated on MHA plates. Microbial colonies were formed and counted after 24 h of incubation. The data presented in the results are the means from three independent assays.

### Drug Resistance Experiment

Resistance development of *E. coli* ATCC 25922 against antibiotics was investigated using a sequential passaging method. Briefly, in the first generation, the MIC value was determined as described previously. After a 24 h incubation, bacterial cells growing in a well with a half-MIC level were harvested and diluted to 0.05 at OD_600_, followed by a 1:10 dilution in fresh MHB. The inoculum was subjected to subsequent passage MIC testing, and the process was repeated for 30 days. The fold change in MIC was plotted against the number of passages.

### Antimicrobial Mechanism Measurement

The mechanism of the designed peptides was measured by fluorescence spectroscopy and electron microscopy as shown before. The fluorescent dyes disc_3_-_5_, β-galactosidase, calcein and BC were used to determine cytoplasmic membrane depolarization and the binding affinities of the peptides to LPS and LTA (Wen et al., 2013; Chou et al., 2019). Briefly, for depolarization, 0.4 μM disc_3_-_5_ was added to the bacteria, which were washed and diluted into an OD600 of 0.05 with 5 mM HEPES buffers (containing 20 mM glucose and 0.1 KCl); for inner membrane permeability, bacteria incubated in LB containing 2% lactose were washed and diluted to an OD600 of 0.05 with 10 mM PBS (pH 7.4) containing 1.5 mM ONPG; for LPS or LTA binding, LPS/LTA and BC were mixed in Tris buffer (50 mM, pH 7.4) with a final concentration of 50 and 5 μg/ml, respectively. Four hours later, the probe mixture was added to 96-well containing peptides with various concentration and incubated for 1 h. The fluorescence levels indicated ROS production, which was reported as important for the apoptosis-like death in bacteria since ROS can destroy all three major classes of macromolecules and compromise cell viability. And we tested the intracellular production of ROS by 2′, 7′-dichlorofluorescin diacetate (DCFH-DA) with bacteria of OD600 = 0.6 and DCFH-DA = 10 μM incubating for 1 h at 37°C (Maurya et al., 2013; Lam et al., 2016). In addition, SEM, TEM, and CLSM were used to obtain direct visualization of bacterial membrane damage (Wang et al., 2018; Chou et al., 2019), and the bacteria samples (OD_600_ = 0.05) were treated with 1 × MIC peptide for half an hour.

### Mouse Model and Cytokine Assay

Female Institute for Cancer Research (ICR) mice (SPF, 20–25 g) were purchased from WeiTonglihua, Co., Ltd. (Beijing, China). Mice were fed and acclimatized as described (Chou et al., 2019). *E. coli* ATCC 25922 was adopted as the pathogenic bacterium for the infection model in this study. In this mouse model, the antimicrobial activity of S7 was determined by colony counting of blood and various organs. Briefly, the mice were divided into four groups (six groups for anti-inflammatory assay) and received intraperitoneal injections of 100 μl *E. coli* (1 × 10^8^ CFU/ml) cells in 0.9% NaCl or only 100 μl 0.9% NaCl as control. 1 h later, 100 μl S7 (5 mg/kg), 100 μl 0.9% NaCl, 100 μl gentamicin (5 mg/kg) or 100 μl 0.9% NaCl, was intraperitoneally injected to the treated or control groups, respectively. And the mice were sacrificed via excessive anesthesia after 6 h post-challenge. The anti-inflammatory effect of S7 was measured by testing the levels of TNF-α, IL-6, and IL-1β in serum using a TNF-α enzyme-linked immunosorbent assay (ELISA) kit, an IL-6 ELISA kit and an IL-1β ELISA kit according to the manufacturer’s instructions (Shanghai Jin Ma Laboratory Equipment, Co., Ltd.).

### Statistical Analysis

The statistical significance of the experimental results was determined by a one-way ANOVA, followed by Duncan’s test. Values of ^∗^*P* < 0.05 were considered statistically significant. GraphPad Prism version 6.0 (San Diego, CA, United States) was used for all statistical analyses.

## Results

### Design and Characterization of the Peptides

The fidelity, purity and molecular weights of the peptides were verified by matrix-assisted laser desorption/ionization time-of-flight mass spectrometry (MALDI-TOF MS; Linear Scientific, Inc., United States) and reverse-phase high-performance liquid chromatography (RP-HPLC), which indicated that the measured molecular weights of the peptides were close to their theoretical molecular weights (Table 1) and that the purities of the peptides were more than 95%, indicating that successful synthesis of the peptides.

**TABLE 1:** Peptides design and their key physicochemical parameters.

Peptide	Sequence	Theoretical MW	Measured MW^a^	Net charge	H^b^
S1	RRWCWWWCWRR-NH2	1780.11	1779.1	5	0.935
S2	RRWYWWWYWRR-NH2	1900.18	1899.16	5	0.830
S3	RRWNWWWNWRR-NH2	1802.04	1801.02	5	0.546
S4	RRWQWWWQWRR-NH2	1830.09	1829.08	5	0.615
S5	RRWTWWWTWRR-NH2	1775.06	1774.8	5	0.703
S6	RRWGWWWGWRR-NH2	1686.92	1686.7	5	0.655
S7	RRWSWWWSWRR-NH2	1747	1746	5	0.648

*^*a*^Molecular weight (MW) was measured by mass spectroscopy (MS). ^*b*^H was the total hydrophobicity (sum of all residue hydrophobicity indices) divided by number of residues and calculated from http://heliquest.ipmc.cnrs.fr/cgi-bin/ComputParams.py.*

### Biocompatibility Assays

**FIGURE 1:**
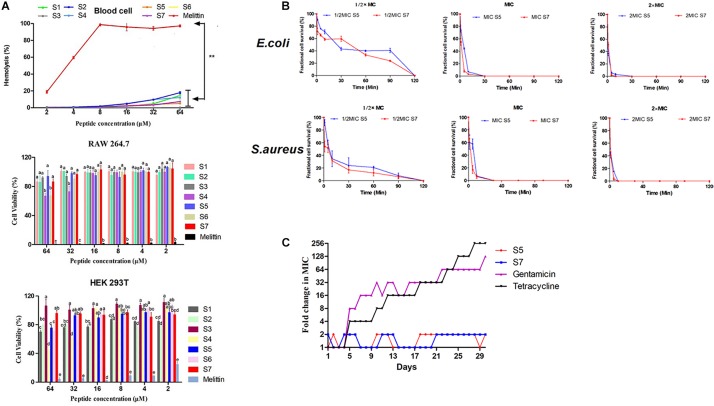
Cell toxicity of the peptides against erythrocytes, RAW264.7 and HEK 293T. Statistically significant differences are noted with different letters (*P* < 0.05) and ** represents significant difference compared with Melittin (*P* <0.01) **(A)**; Fractional microbial survival of *Escherichia coli* ATCC 25922 and *Staphylococcus aureus* ATCC 29213 treated with S5 and S7 **(B)**; Resistance development in the presence of sub-MIC concentration of S5, S7, gentamicin, and tetracycline **(C)**.

High biocompatibility is a prerequisite for any bioactive agents to break through clinical application bottlenecks. Thus, the activities of all designed peptides against fresh, healthy human erythrocytes, human embryonic kidney 293T cells and murine macrophage cell line RAW 264.7 cells were first assessed, as shown in Figure 1A, all the designed peptides showed significantly lower hemolytic activities than the typical peptide melittin (*P* < 0.01). The peptide cytotoxicity against RAW 264.7 cells and HEK 293T cells also confirmed that the cell viabilities after exposure to these peptides were higher than 70% (except for S4 and S6 at 64 μM), which were significantly higher than those after exposure to melittin (*P* < 0.05).

### Antimicrobial Activity Assay

The antibacterial activities of these peptides against *Staphylococcus epidermidis*, *Staphylococcus aureus*, *Escherichia coli*, *Pseudomonas aeruginosa* and *Lactobacillus* are shown in Table 2, S5 and S7 showed the best activity and specificity among this series, with geometric means (GMs) of 2.24 and 3.17 μM against pathogens, respectively, and more than 64 μM against *Lactobacillus.*

**TABLE 2:** The MICs of the peptides against pathogenic or probiotic bacteria.

	MICs (μ M)											
Peptides	*E. coli* 25922	*E. coli* UB 1005	*P. aeruginosa* 27853	*S. epidermidis* 12228	*S. aureus* 29213	MRSA* 43300	*L. rhamnosus 1.9025*	*L. rhamnosus* 1.0386	*L. rhamnosus* 7469	*L. rhamnosus* 1.0911	*L. rhamnosus* 1.0385	*L. plantarum 8014*
S1	>64	16	8	8	8	4	>64	>64	>64	>64	16	8
S2	32	32	8	8	8	8	>64	16	>64	>64	16	4
S3	>64	>64	>64	>64	>64	>64	>64	>64	>64	>64	>64	>64
S4	4	4	4	4	4	4	>64	64	>64	>64	16	8
S5	2	2	4	2	1	4	>64	>64	>64	>64	>64	>64
S6	4	2	8	>64	16	16	>64	>64	>64	>64	>64	>64
S7	4	2	4	4	4	2	>64	>64	>64	>64	>64	>64

*Methicillin-resistant *Staphylococcus aureus*.

### Time-Kill Curve Assay

Based on the above results, the ability of the peptides S5 and S7 to kill the model pathogens *E. coli* 25922 and *S. aureus* 29213 was further investigated by analyzing fractional cell survival upon peptide treatment at the 1/2, 1, and 2 MIC at various exposure times. Our results (Figure 1B) showed that the sterilization rate was dependent on the concentration of the peptide, with higher concentrations having faster rates.

### Salt and Serum Susceptibility Assays

The susceptibility of antimicrobial peptides to salt or a serum environment has prevented many potential peptides from achieving clinical application. Thus, the antimicrobial activities of S5 and S7 against *E. coli* 25922 and *S. aureus* 29213 in the presence of physiological concentrations of different salts or a serum environment were further evaluated. While their antimicrobial activities against *S. aureus* 29213 were retained in the presence of all salts, S5 and S7 retained their antimicrobial activity against *E. coli* 25922 in the presence of most salts except Fe^3+^ and Ca^2+^, with Fe^3+^ increasing the MICs of S5 and S7 up to 8- and 4-fold, respectively, and Ca^2+^ increasing the MIC of S5 up to 4-fold. S5 and S7 showed compromised activity in the serum environment, and the MICs of S5 and S7 increased up to 16- and 4-fold in the presence of 50% serum, respectively (Table 3).

**TABLE 3:** The MIC^a^ values of the peptides against *E. coli* ATCC 25922 and *S. aureus* ATCC 29213 in the presence of physiological salts or serum environment.

Peptide	Control^†^	NaCl^†^	KCl^†^	MgCl_2_^†^	NH_4_Cl^†^	ZnCl_2_^†^	FeCl_3_^†^	CaCl_2_^†^	50% of serum	25% of serum	12.5% of serum
***Gram-negative strain E. coli* ATCC 25922**											
S5	2	4	4	1	4	1	16	8	32	32	32
S7	4	8	4	8	4	1	16	4	16	16	16
***Gram-positive strain S. aureus* ATCC 29213**											
S5	1	4	2	1	2	1	4	2	32	8	8
S7	4	4	2	2	2	1	4	2	16	8	4

*^†^The final concentrations of NaCl, KCl, NH_4_Cl, MgCl_2_, CaCl_2_, ZnCl_2_, and FeCl_3_ were 150 mM, 4.5 mM, 6 μM, 1 mM, 2 mM, 8 μM, and 4 μM, respectively, and the control MIC values were determined in the absence of these physiological salts. The data were derived from representative value of three independent experimental trials.*

### Drug Resistance Assay

Drug resistance was induced by treating *E. coli* 25922 with S5 and S7, with gentamicin and tetracycline as controls. As shown in Figure 1C, compared with gentamicin and tetracycline, which induced rapid resistance acquisition, S5 and S7 displayed relatively constant MIC values against these bacterial strains throughout the experiment following serial passaging of bacterial cells in the presence of sub-MICs of the antimicrobial peptides, suggesting that it is difficult for bacteria to acquire resistance to S5 and S7.

### Antibacterial Mechanism Study

In addition to these potential application effect assays, S5 and S7 were further studied for their activity mechanism for killing the model bacteria *E. coli* 25922 and *S. aureus* 29213.

**FIGURE 2:**
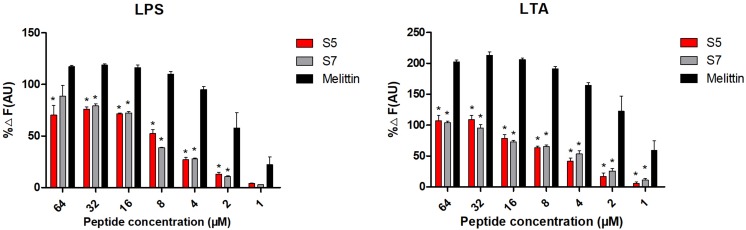
Antibacterial mechanism study Combination of lipopolysaccharide (LPS from *E. coli*) and lipoteichoic acid (LTA from *S. aureus*) with peptides (S5, S7 and Melittin). * represents significant difference compared with Melittin (*P* < 0.05).

Based upon molecular components of gram-positive and gram-negative bacterial membranes, binding to LPS or LTA located on the outer membrane (OM) was speculated to be the first step of S5 and S7 acting on the bacteria. The experimental result (Figure 2) showed that S5 and S7 could bind to LPS or LTA in a dose-dependent manner. However, compared with the broad-spectrum peptide melittin, S5 and S7 showed a significant reduction in fluorescence intensity (*P* < 0.05), indicating a lower affinity of these two peptides to LPS or LTA than that of melittin.

**FIGURE 3:**
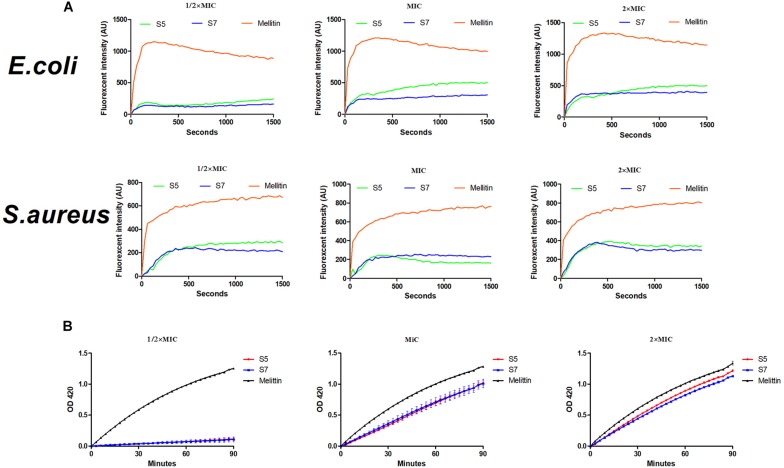
Interaction of Cytoplasmic membrane with peptides (S5, S7 and Melittin) against *E. coli* ATCC 25922 and *S. aureus* 29213 **(A)** and hydrolysis of ONPG due to release of cytoplasmic β-galactosidase of *E. coli* UB 1005 treated by 1/2, 1, and 2×MIC peptides were measured spectroscopically at absorbance of 420 nm as a function of time **(B)**.

Subsequently, cytoplasmic membrane perturbation and permeability were further assessed by measuring the cytoplasmic membrane depolarization level and the release of cytoplasmic β-galactosidase. The results (Figure 3A) showed that membrane potential changes in *E. coli* ATCC 25922 and *S. aureus* 29213 induced by the 1/2, 1, and 2 MIC of S5 and S7 were time- and dose-dependent within 1500 s but they were still lower than those induced by melittin. Additionally, the results of the release of cytoplasmic β-galactosidase by *E. coli* UB 1005 (Figure 3B) showed that S5 and S7 induced a rapid release of cytoplasmic β-galactosidase at the 1 and 2 MIC. The release of cytoplasmic β-galactosidase induced by S5 and S7 at their MICs were significantly lower than that induced by melittin (^∗∗^*P* < 0.01), but they all show similarly cytoplasmic membrane permeability at their 2 MICs (*P* > 0.01).

**FIGURE 4:**
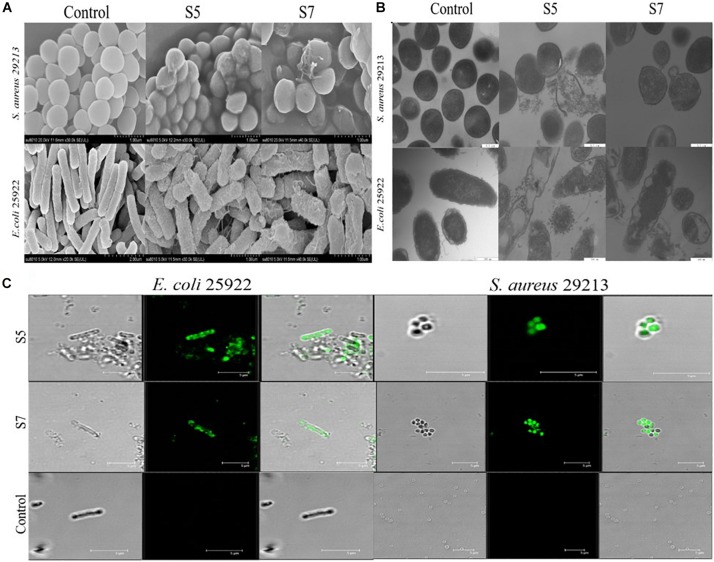
Membrane damage effect of *E. coli* ATCC 25922 and *S. aureus* 29213 treated by S5 and S7 was observed by SEM and TEM **(A,B)**; Combination of *E. coli* 25922 and *S. aureus* 29213 with FITC-labeled peptides (S5 and S7) was observed using confocal fluorescence microscope **(C)**.

**FIGURE 5:**
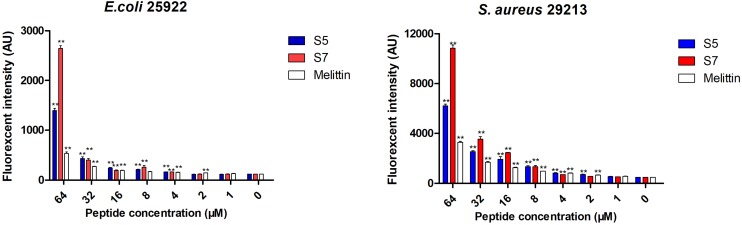
The effects of S5, S7 and Melittin on the levels of ROS. Data represents the mean ± SEM. ** means *P* < 0.01 compared with the untreated cells.

Finally, the visualized cell morphology and intracellular ultrastructural alterations of cells treated with S5 and S7 were observed by SEM, TEM, and CLSM. The SEM results (Figure 4A) showed that a control bacterial membrane was intact and smooth, while the bacterial membranes treated with S5 and S7 were roughened and blebby, suggesting that S5 and S7 could induce obvious pore formation and membrane destruction. Observation by TEM (Figure 4B) further suggested that S5 and S7 resulted in obvious alterations of membrane morphology, loss of intracellular contents, and obvious clear areas compared with the dense internal structures of the control group. Furthermore, FITC-labeled peptides were used to monitor peptide localization by Confocal Laser-Scanning Microscopy, and Figure 4C showed that S5 or S7 presented green fluorescent signal distribution around the *E. coli* and *S. aureus* cell surface, indicating that they targeted the cell membrane surface. In addition to these membrane interaction assays; ROS production was tested to determine the apoptosis-like death in bacteria. Figure 5 showed that all the tested peptides could induce significant fluorescence increase in the bacteria cells at concentrations higher than MIC.

### *In vivo* Efficacies of the Selective Peptide

**FIGURE 6:**
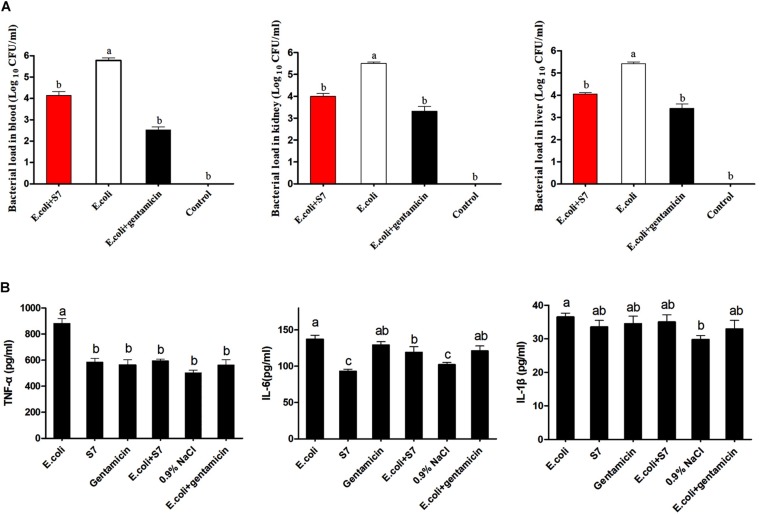
Bacterial load in blood and major mouse organs **(A)**. Statistically significant differences are noted with different letters (*P* < 0.05); Effect of S7 on the TNF- α (Left), IL- 6 (Middle), and IL- 1β (Right) levels in the serum (*n* = 6) **(B)**. Data represents the mean ± SEM. Means with different letters represent significantly different at *P* < 0.05 by Duncan’s test.

To further confirm the *in vivo* antimicrobial effect of the novel designed antimicrobial peptide, S7 was selected to treat the mice infected with *E. coli* ATCC 25922, and the activity was demonstrated by counting bacterial load distribution. And the bacterial colony distribution in the blood, kidney and liver showed that the number of colonies in control group (only injected with twice 100 μl 0.9% NaCl) was none, and the number of colonies in the S7 + *E. coli* and gentamicin + *E. coli* groups were significantly decreased compared with that in the *E. coli* group (*P* < 0.05) after 6 h post-treatment (Figure 6A). In addition, the inflammatory cytokines were further tested to expose the anti-inflammatory effect of S7, and the levels of the inflammatory cytokines (TNF-α, IL-6, and IL-1β) shown that the serum levels of TNF-α and IL-6 in S7 + *E. coli* group were decreased significantly compared with those of *E. coli* group (*P* < 0.05) but the S7 + *E. coli* group showed no significant difference in the level of IL-1β compared with the *E. coli* group (*P* > 0.05) (Figure 6B).

## Discussion

Tryptophan (W), which is thought to be able to cross the blood–brain barrier (BBB) in a free form, has been demonstrated to be a non-toxic exogenously supplied amino acid, with a median lethal dose (LD50) as high as 1.6 g/kg in rats, corresponding to ∼ 1.4 g/kg in humans (Richard et al., 2009; Fernstrom, 2012). Also, the indole ring of tryptophan prefers to interact with a polar-polar interface, and the indole NH being close to a lipid carbonyl ester leads W-containing peptides to be powerful and safe promising antibiotic candidates with different beneficial effects (Dixon et al., 2003; Lee et al., 2018). Paired consecutive arginine residues were appended to enhance electrostatic interactions by strong bidentate H-bonds with phosphate moieties from two lipid headgroups and electrostatic cation-π interactions with aromatic residues (Mecozzi et al., 1996; Gallivan and Dougherty, 1999; Torcato et al., 2013). Thus, sequences rich in Arg and Trp showed higher antimicrobial activity by forming hydrogen bonds as these peptides associate with membrane components (Liu et al., 2007). Moreover, it is well-known that probiotics also take an important role in killing bacteria, modulating intestinal barrier and modulating immune system. Our previous selective-template against Gram-negative has proved that antimicrobial-spectrum of a given peptide could be modified via replacing hydrophilic amino acids (Chou et al., 2019). Thus, the final peptide series provided a comparative picture of the effect of different hydrophilic amino acids on the antimicrobial spectrum, depending on the triplet-tryptophan-pivot design.

The toxicity of synthetic peptides against host cells is frequently believed to be the chief limitation of using AMPs as novel therapeutics. The biocompatibility assay results revealed that these triplet-tryptophan-pivot peptides are safe for use in mammals due to their proper hydrophobicity, which was determined to be the most critical structural factor that determines the toxicity of AMPs (Schmidtchen et al., 2014). In addition, different cell survival rates within the triplet-tryptophan-pivot groups demonstrated that hydrophilic amino acids also significantly affected biocompatibility, which might be due to hydrophilic amino acids changing not only the total hydrophobicity but also the folding structure of the peptides.

Antimicrobial peptides exhibit varying antimicrobial activities against microbes by acting on the target cell membranes via a non-receptor-mediated pathway, making it possible to replace traditional antibiotics; thus, selective activity of peptides is thought to be a challenge for the application of AMPs as future biomaterials without damaging natural probiotic levels. Previous reports demonstrated that the antimicrobial activity of peptides was related to positive charges, amino acid composition, hydrophobicity, residue distribution, and secondary structure (Khara et al., 2017; Amso and Hayouka, 2019). In our study, the surprise result that most of these triplet-tryptophan-pivot peptides (except for S3) possessed efficient antimicrobial activity against pathogenic bacteria, including both Gram-negative and Gram-positive ones, while showing no effect on the tested probiotics even at the highest tested concentration (64 μM) demonstrated that positive charge and hydrophobic amino acid distribution are the key factors in determining the bioactivity of peptides, and this short template containing 4 Arg and 5 Trp residues showed high potential selectivity of antimicrobial agents for maintaining microecological balance (Chai et al., 2019). Besides, these seven sequences showed varying degrees of antibacterial activities against tested bacteria, which indicated hydrophilic amino acids a vital role in adjusting the antibacterial spectrum. First, S1 and S2, which had higher value of H (H represented the total hydrophobicity (sum of all residue hydrophobicity indices) divided by number of residues), showed lower antimicrobial activity against the Gram-negative *E. coli* demonstrated that the hydrophobicity of cysteine (C) or tyrosine (Y) was too high for this sequence, which might prevent these two peptides from transporting through non-specific transmembrane water-filled channels (or pores) of outer membrane of Gram-negative (Novikova and Solovyeva, 2009). Additionally, the non-activity of S3 indicated that the hydrophilicity of S3 might be not enough to break down the bacterial membrane because the high hydrophilicity of asparagine (N) decreased the total hydrophobicity of S3. The comparison of S5 and S7 with S6 demonstrated that this selectivity might be related to the folding structure of the final sequence affected by Glycine (G), which was following that a glycine-rich composition could facilitate flexibility or dynamicity and induced specifically antibacterial activity against Gram-negative bacteria (Chou et al., 2019). Thus, the comparison of seven hydrophilic amino acid peptides that had the same positive charge and hydrophobic amino acid composition indicated that the antimicrobial spectrum can be regulated by selecting different types of hydrophilic amino acids, which was in accordance with that the hydrophilic–hydrophobic balance of antimicrobial polymer significantly affects the interactions between the micelles and cell membrane (Cao et al., 2018).

Electrostatic adsorption with the negatively charged surface of a microorganism is the basis for the selectivity of cationic peptides, which will be inhibited by charge screening effects or competitive binding of some free ions, such as monovalent cations (Na^+^ and K^+^), divalent cations (Ca^2+^ and Mg^2+^) and multivalent cations (Fe^3+^) in the surrounding medium. In contrast, it was also reported that additional divalent cations could enhance the adsorption capacity of AMPs to the bacterial surface (Fedders et al., 2008; Aquila et al., 2013). Thus, it was hypothesized that the proportion of AMP and cation concentrations was increased. In our study, both S5 and S7 maintained their antimicrobial activities against pathogenic bacteria in the presence of salt (except for Fe^3+^) due to having enough positive charge or Trp composition, which could induce deep penetration of the peptides into the bacterial membrane and large membrane-bound surface area (Yu et al., 2013; Saravanan et al., 2014). To further confirm whether S5 and S7 could prevent or slow bacteria from becoming resistant, an evolution study of *E. coli* ATCC 25922 with sequential passaging was performed, and these drug resistance results forcefully proved that S5 and S7 are not affected by traditional mechanisms of bacterial resistance.

Antimicrobial peptides, which are regarded as the antibiotic substitute with the most potential for being not affected by traditional mechanisms of bacterial resistance, lead to cell death by physical disruption of the pathogen’s membrane and subsequent leakage of cytoplasmic components, which greatly reduces the development of resistance against drugs (Makovitzki et al., 2008; Wimley, 2010; Teixeira et al., 2012; Jiang et al., 2017). Thus, the antibacterial mechanisms of S5 and S7 were further confirmed by testing the interaction of peptides with lipopolysaccharide (LPS), lipoteichoic acid (LTA) and the cytoplasmic membrane (CM), the major components related to the mechanism of action of antimicrobial peptides. Negatively-charged lipopolysaccharides (LPS) or lipoteichoic acids (LTA) may interact with peptides via charge residues, which could attract antimicrobial peptides to increase the hydrophobic interaction, thereby increasing their antimicrobial effects (Papo and Shai, 2005; Bucki and Janmey, 2006; Ong et al., 2013). In this study, S5 and S7 showed lower binding affinity to LPS and LTA than to that of the broad-spectrum antimicrobial peptide melittin. Followed by the lower cytoplasmic membrane depolarization and the release of cytoplasmic β-galactosidase results of S5 and S7 compared with melittin. It was suggested that S5 and S7 could destroy the integrity of the cell membrane of the pathogenic bacteria, and the selectivity of S5 and S7 against pathogenic bacteria rather than probiotic bacteria might be due to the slight membrane damage effect. SEM, TEM, and CLSM results directly demonstrated that S5 and S7 can bind to bacterial membrane and cause obvious damage to the bacterial membrane, including blebbing of the outer membrane, pore formation in the cell membrane and leakage of intracellular contents, suggesting that S5 and S7 exert their bactericidal effects through mainly the action associated with membrane disruption. Additionally, there are indications that S5 and S7 could also induce accumulation of reactive oxygen species (ROS) in *E. coli* and *S. aureus*. These bactericidal mechanisms have little or no potential for selection of resistance, as the development of resistance would require an extraordinary number of mutations involving genes coding for pathways that generate bacterial cell wall architecture (Rodrigues De Almeida et al., 2019).

To date, most AMPs still display strong antimicrobial activity *in vitro* with slight or no activity *in vivo*. Thus, the application potential of the selected peptide was tested using a bacteria-infected mouse model. In this model, treatment was performed 1 h after inoculation, as 1 h is sufficient to allow bacteria to disseminate throughout the host (Zhang et al., 2015; Liu et al., 2017). Our bacterial load data showed that S7 had effective therapeutic activity, reducing the bacterial load in the mice compared with that the *E. coli*-injected mice. In addition, the inflammatory cytokine level in serum was also tested because it reflects the outcome and severity of the infection (Xing et al., 1998; Lopez-Castejon and Brough, 2011; Conrad et al., 2018). The result that the release of TNF-α and IL-6 was significantly decreased after S7 treatment suggested that the peptide might suppress the development of a cytokine storm via a rapid bactericidal effect *in vivo*, demonstrating that S7 is a promising therapeutic agent against bacterial infections in the clinical settings.

In this study, we proposed a series of triplet-tryptophan-pivot peptides and presented evidences of the effect that different hydrophilic amino acids had on peptide antimicrobial spectrum. These peptides showed high biocompatibility and retained antimicrobial activity in the presence of salt due to the high composition of tryptophan. The higher activity of S5 and S7 against pathogenic bacteria vs. probiotic bacteria showed the benefit of choosing threonine and serine. The membrane destruction of these peptides demonstrated that they have great potential to replace antibiotics with little potential for the development of resistance and long-lasting protective effects. Meanwhile, S7 displayed strong effective antimicrobial potency in a mouse model. Taken together, our studies not only analyzed the functions of hydrophilic amino acids on antimicrobial activity but also expanded the design method to develop bacteria-selective peptides.

## Data Availability Statement

The raw data supporting the conclusions of this article will be made available by the authors, without undue reservation, to any qualified researcher.

## Ethics Statement

Ethical review and approval was not required for the study on human participants in accordance with the local legislation and institutional requirements. The patients/participants provided their written informed consent to participate in this study. The animal study was reviewed and approved by the Animal Care and Use Committee of Northeast Agricultural University.

## Author Contributions

SC and AS designed the research. QL, ZN, LS, JZL, and JWL performed the research. ZW and JWL performed analyses. All the authors have read and approved the manuscript.

## Conflict of Interest

The authors declare that the research was conducted in the absence of any commercial or financial relationships that could be construed as a potential conflict of interest.

## References

[B1] Aguilar-Toalá J. E. Hernández-Mendoza A. González-Córdova A. F. Vallejo-Cordoba B. Liceaga A. M. (2019). Potential role of natural bioactive peptides for development of cosmeceutical skin products. *Peptides* 122:170170. 10.1016/j.peptides.2019.170170 31574281

[B2] Amso Z. Hayouka Z. (2019). Antimicrobial random peptide cocktails: a new approach to fight pathogenic bacteria. *Chem. Commun.* 55 2007–2014. 10.1039/c8cc09961h 30688322

[B3] Aquila M. Benedusi M. Koch K. W. Dell’Orco D. Rispoli G. (2013). Divalent cations modulate membrane binding and pore formation of a potent antibiotic peptide analog of alamethicin. *Cell Calcium* 53 180–186. 10.1016/j.ceca.2012.11.012 23261317

[B4] Barbosa M. Costa F. Monteiro C. Duarte F. Martins M. C. L. Gomes P. (2019). Antimicrobial coatings prepared from Dhvar-5-click-grafted chitosan powders. *Acta Biomater.* 84 242–256. 10.1016/j.actbio.2018.12.001 30528610

[B5] Bubnov R. V. Babenko L. P. Lazarenko L. M. Mokrozub V. V. Spivak M. Y. (2018). Specific properties of probiotic strains: relevance and benefits for the host. *EPMA J.* 9 205–223. 10.1007/s13167-018-0132-z 29896319 PMC5972142

[B6] Bucki R. Janmey P. A. (2006). Interaction of the gelsolin-derived antibacterial PBP 10 peptide with lipid bilayers and cell membranes. *Antimicrob. Agents Chemother.* 50 2932–2940. 10.1128/AAC.00134-06 16940084 PMC1563552

[B7] Cao B. Xiao F. Xing D. Hu X. (2018). Polyprodrug antimicrobials: remarkable membrane damage and concurrent drug release to combat antibiotic resistance of methicillin-resistant *Staphylococcus aureus*. *Small* 14 1–13. 10.1002/smll.201802008 30118562

[B8] Chai T. T. Tan Y. N. Ee K. Y. Xiao J. Wong F. C. (2019). Seeds, fermented foods, and agricultural by-products as sources of plant-derived antibacterial peptides. *Crit. Rev. Food Sci. Nutr.* 59 1–16. 10.1080/10408398.2018.1561418 30663883

[B9] Chou S. Shao C. Wang J. Shan A. Xu L. Dong N. (2016). Short, multiple-stranded β-hairpin peptides have antimicrobial potency with high selectivity and salt resistance. *Acta Biomater.* 30 78–93. 10.1016/j.actbio.2015.11.002 26546414

[B10] Chou S. Wang J. Shang L. Akhtar M. U. Wang Z. Shi B. (2019). Short, symmetric-helical peptides have narrow-spectrum activity with low resistance potential and high selectivity. *Biomater. Sci.* 7 2394–2409. 10.1039/c9bm00044e 30919848

[B11] Conrad C. Di Domizio J. Mylonas A. Belkhodja C. Demaria O. Navarini A. A. (2018). TNF blockade induces a dysregulated type i interferon response without autoimmunity in paradoxical psoriasis. *Nat. Commun.* 9:25. 10.1038/s41467-017-02466-4 29295985 PMC5750213

[B12] Dixon G. Nolan J. McClenaghan N. Flatt P. R. Newsholme P. (2003). A comparative study of amino acid consumption by rat islet cells and the clonal beta-cell line BRIN-BD11 - the functional significance of L-alanine. *J. Endocrinol.* 179 447–454. 10.1677/joe.0.1790447 14656214

[B13] Dong N. Wang C. Zhang T. Zhang L. Xue C. Feng X. (2019). Bioactivity and bactericidal mechanism of histidine-rich β -hairpin peptide against Gram-negative bacteria. *Int. J. Mol. Sci.* 20:3954. 10.3390/ijms20163954 31416220 PMC6718988

[B14] Fedders H. Michalek M. Grötzinger J. Leippe M. (2008). An exceptional salt-tolerant antimicrobial peptide derived from a novel gene family of haemocytes of the marine invertebrate *Ciona intestinalis*. *Biochem. J.* 416 65–75. 10.1042/BJ20080398 18598239

[B15] Fernstrom J. D. (2012). Effects and side effects associated with the non-nutritional use of tryptophan by humans. *J. Nutr.* 142 2236S–2244S. 10.3945/jn.111.157065 23077193

[B16] Fuente-nunez C. De Torres M. D. T. Mojica F. J. M. Lu T. K. (2017). ScienceDirect Next-generation precision antimicrobials: towards personalized treatment of infectious diseases. *Curr. Opin. Microbiol.* 37 95–102. 10.1016/j.mib.2017.05.014 28623720 PMC5669808

[B17] Gallivan J. P. Dougherty D. A. (1999). Cation-π interactions in structural biology. *Proc. Natl. Acad. Sci. U.S.A.* 96 9459–9464. 10.1073/pnas.96.17.9459 10449714 PMC22230

[B18] Jiang Y. Zheng W. Kuang L. Ma H. Liang H. (2017). Hydrophilic phage-mimicking membrane active antimicrobials reveal nanostructure-dependent activity and selectivity hydrophilic phage-mimicking membrane active antimicrobials reveal nanostructure-dependent activity and selectivity. *ACS Infect. Dis.* 3 676–687. 10.1021/acsinfecdis.7b00076 28758395

[B19] Khara J. S. Obuobi S. Wang Y. Hamilton M. S. Robertson B. D. Newton S. M. (2017). Disruption of drug-resistant biofilms using de novo designed short α-helical antimicrobial peptides with idealized facial amphiphilicity. *Acta Biomater.* 57 103–114. 10.1016/j.actbio.2017.04.032 28457962

[B20] Lam S. J. O’Brien-Simpson N. M. Pantarat N. Sulistio A. Wong E. H. H. Chen Y. Y. (2016). Combating multidrug-resistant Gram-negative bacteria with structurally nanoengineered antimicrobial peptide polymers. *Nat. Microbiol.* 1:16162. 10.1038/nmicrobiol.2016.162 27617798

[B21] Lee M. Y. Park S. C. Jung M. Shin M. K. Kang H. L. Baik S. C. (2018). Cell-selectivity of tryptophan and tyrosine in amphiphilic α-helical antimicrobial peptides against drug-resistant bacteria. *Biochem. Biophys. Res. Commun.* 505 478–484. 10.1016/j.bbrc.2018.09.095 30268502

[B22] Liu B. Huang H. Yang Z. Liu B. Gou S. Zhong C. (2017). Design of novel antimicrobial peptide dimer analogues with enhanced antimicrobial activity in vitro and in vivo by intermolecular triazole bridge strategy. *Peptides* 88 115–125. 10.1016/j.peptides.2016.12.016 28040477

[B23] Liu Z. Brady A. Young A. Rasimick B. Chen K. Zhou C. (2007). Length effects in antimicrobial peptides of the (RW)n series. *Antimicrob. Agents Chemother.* 51 597–603. 10.1128/AAC.00828-06 17145799 PMC1797765

[B24] Lopez-Castejon G. Brough D. (2011). Understanding the mechanism of IL-1β secretion. *Cytokine Growth Factor Rev.* 22 189–195. 10.1016/j.cytogfr.2011.10.001 22019906 PMC3714593

[B25] Ma Z. Yang J. Han J. Gao L. Liu H. Lu Z. (2016). Insights into the antimicrobial activity and cytotoxicity of engineered α-helical peptide amphiphiles insights into the antimicrobial activity and cytotoxicity of engineered α-helical peptide amphiphiles. *J. Med. Chem.* 24 10946–10962. 10.1021/acs.jmedchem.6b00922 28002968

[B26] Makovitzki A. Baram J. Shai Y. (2008). Antimicrobial lipopolypeptides composed of palmitoyl di- and tricationic peptides: *in vitro* and in vivo activities, self-assembly to nanostructures, and a plausible mode of action. *Biochemistry* 47 10630–10636. 10.1021/bi8011675 18783248

[B27] Maurya I. K. Thota C. K. Sharma J. Tupe S. G. Chaudhary P. Singh M. K. (2013). Mechanism of action of novel synthetic dodecapeptides against *Candida albicans*. *Biochim. Biophys. Acta - Gen. Subj.* 1830 5193–5203. 10.1016/j.bbagen.2013.07.016 23876294

[B28] Mecozzi S. West A. P. Dougherty D. A. (1996). Cation-π interactions in aromatics of biological and medicinal interest: electrostatic potential surfaces as a useful qualitative guide. *Proc. Natl. Acad. Sci. U.S.A.* 93 10566–10571. 10.1073/pnas.93.20.10566 8855218 PMC38193

[B29] Mishra B. Wang G. (2012). Ab initio design of potent Anti-MRSA peptides based on database part I: experimental section. *J. Chem. Soc.* 134 12426–12429. 10.1021/ja305644e 22803960 PMC3412535

[B30] Novikova O. D. Solovyeva T. F. (2009). Nonspecific porins of the outer membrane of Gram-negative bacteria: structure and functions. *Biochem. Suppl. A Membr. Cell Biol.* 3 3–15. 10.1134/S1990747809010024

[B31] Ong Z. Y. Gao S. J. Yang Y. Y. (2013). Short synthetic β -sheet forming peptide amphiphiles as broad spectrum antimicrobials with antibiofilm and endotoxin neutralizing capabilities. *Adv. Funct. Mater.* 23 3682–3692. 10.1002/adfm.201202850

[B32] Papo N. Shai Y. (2005). A molecular mechanism for lipopolysaccharide protection of gram-negative bacteria from antimicrobial peptides. *J. Biol. Chem.* 280 10378–10387. 10.1074/jbc.M412865200 15632151

[B33] Richard D. M. Dawes M. A. Mathias C. W. Acheson A. Hill-Kapturczak N. Dougherty D. M. (2009). L-tryptophan: basic metabolic functions, behavioral research and therapeutic indications. *Int. J. Tryptophan Res.* 2 45–60. 10.4137/ijtr.s2129 20651948 PMC2908021

[B34] Rodrigues De Almeida N. Han Y. Perez J. Kirkpatrick S. Wang Y. (2019). Design, synthesis, and nanostructure-dependent antibacterial activity of cationic peptide amphiphiles. *ACS Appl. Mater. Interfaces* 11 2790–2801. 10.1021/acsami.8b17808 30588791 PMC7199185

[B35] Saravanan R. Li X. Lim K. Mohanram H. Peng L. Mishra B. (2014). Design of short membrane selective antimicrobial peptides containing tryptophan and arginine residues for improved activity, salt-resistance, and biocompatibility. *Biotechnol. Bioeng.* 111 37–49. 10.1002/bit.25003 23860860

[B36] Schmidtchen A. Pasupuleti M. Malmsten M. (2014). Effect of hydrophobic modifications in antimicrobial peptides. *Adv. Coll. Interface Sci.* 205 265–274. 10.1016/j.cis.2013.06.009 23910480

[B37] Shao C. Li W. Tan P. Shan A. Dou X. Ma D. (2019). Symmetrical modification of minimized dermaseptins to extend the spectrum of antimicrobials with endotoxin neutralization potency. *Int. J. Mol. Sci.* 20:1417. 10.3390/ijms20061417 30897850 PMC6470953

[B38] Singh M. Mukhopadhyay K. (2014). Alpha-melanocyte stimulating hormone: an emerging anti-inflammatory antimicrobial peptide. *Biomed. Res. Int.* 2014:874610. 10.1155/2014/874610 25140322 PMC4130143

[B39] Teixeira V. Feio M. J. Bastos M. (2012). Role of lipids in the interaction of antimicrobial peptides with membranes. *Prog. Lipid Res.* 51 149–177. 10.1016/j.plipres.2011.12.005 22245454

[B40] Tenover F. C. (2006). Mechanisms of antimicrobial resistance in bacteria. *Am. J. Infect. Control* 34(5 Suppl. 1), S3–S10. 10.1016/j.ajic.2006.05.219 16813980

[B41] Torcato I. M. Huang Y. H. Franquelim H. G. Gaspar D. Craik D. J. Castanho M. A. R. B. (2013). Design and characterization of novel antimicrobial peptides, R-BP100 and RW-BP100, with activity against Gram-negative and Gram-positive bacteria. *Biochim. Biophys. Acta Biomembr.* 1828 944–955. 10.1016/j.bbamem.2012.12.002 23246973

[B42] Torres M. D. T. Sothiselvam S. Timothy K. de Fuente-nunez C. (2019). Peptide design principles for antimicrobial applications. *J. Mol. Biol.* 431 3547–3567. 10.1016/j.jmb.2018.12.015 30611750

[B43] Wang J. Chou S. Yang Z. Yang Y. Wang Z. Song J. (2018). Combating drug-resistant fungi with novel imperfectly amphipathic palindromic peptides. *J. Med. Chem.* 61 3889–3907. 10.1021/acs.jmedchem.7b01729 29648811

[B44] Wen Y. L. Wu B. J. Kao P. H. Fu Y. S. Chang L. S. (2013). Antibacterial and membrane-damaging activities of β-bungarotoxin B chain. *J. Pept. Sci.* 19 1–8. 10.1002/psc.2463 23136049

[B45] Wimley W. C. (2010). Describing the mechanism of antimicrobial peptide action with the interfacial activity model. *ACS Chem. Biol.* 5 905–917. 10.1021/cb1001558 20698568 PMC2955829

[B46] Xing Z. Gauldie J. Cox G. Baumann H. Jordana M. Lei X. F. (1998). IL-6 is an antiinflammatory cytokine required for controlling local or systemic acute inflammatory responses. *J. Clin. Invest.* 101 311–320. 10.1172/JCI1368 9435302 PMC508569

[B47] Yu H. Y. Yip B. S. Tu C. H. Chen H. L. Chu H. L. Chih Y. H. (2013). Correlations between membrane immersion depth, orientation, and salt-resistance of tryptophan-rich antimicrobial peptides. *Biochim. Biophys. Acta Biomembr.* 1828 2720–2728. 10.1016/j.bbamem.2013.07.020 23896553

[B48] Zanutto-Elgui M. R. Vieira J. C. S. Prado D. Z. D. Buzalaf M. A. R. Padilha P. D. M. Elgui de Oliveira D. (2019). Production of milk peptides with antimicrobial and antioxidant properties through fungal proteases. *Food Chem.* 278 823–831. 10.1016/j.foodchem.2018.11.119 30583449

[B49] Zarena D. Mishra B. Lushnikova T. Wang F. Wang G. (2017). The π configuration of the WWW motif of a short trp-rich peptide is critical for targeting bacterial membranes, disrupting preformed biofilms, and killing methicillin-resistant *Staphylococcus aureus*. *Biochemistry* 56 4039–4043. 10.1021/acs.biochem.7b00456 28731688 PMC5603908

[B50] Zhang Q. Xu Y. Wang Q. Hang B. Sun Y. Wei X. (2015). Potential of novel antimicrobial peptide P3 from bovine erythrocytes and its analogs to disrupt bacterial membranes In Vitro and display activity against drug-resistant bacteria in a mouse model. *Antimicrob. Agents Chemother.* 59 2835–2841. 10.1128/AAC.04932-14 25753638 PMC4394822

[B51] Zhao J. Zhao C. Liang G. Zhang M. Zheng J. (2013). Engineering antimicrobial peptides with improved antimicrobial and hemolytic activities. *J. Chem. Inf. Model.* 53 3280–3296. 10.1021/ci400477e 24279498

[B52] Zhu X. Ma Z. Wang J. Chou S. Shan A. (2014). Importance of tryptophan in transforming an amphipathic peptide into a *Pseudomonas aeruginosa*-targeted antimicrobial peptide. *PLoS One* 9:e0114605. 10.1371/journal.pone.0114605 25494332 PMC4262413

